# The effect of family-centered empowerment model on burden of care in parents and blood glucose level of children with type I diabetes family empowerment on burden of care and HbA1C

**DOI:** 10.1186/s12912-023-01375-w

**Published:** 2023-06-20

**Authors:** Samira Rostaminasab, Monirsadat Nematollahi, Yones Jahani, Roghayeh Mehdipour-Rabori

**Affiliations:** 1 Nursing Research Center, Kerman University of Medical Sciences, Kerman, Iran; 2 Department of Pediatrics and Neonatal Intensive Nursing, Nursing Research Center, Kerman University of Medical Sciences, Kerman, Iran; 3 Modeling in Health Research Center, Institute for Futures Studies in Health, Kerman University of Medical Sciences, Kerman, Iran; 4 Department of Medical-Surgical Nursing, Nursing Research Center, Kerman University of Medical Sciences, Kerman, Iran

**Keywords:** Diabetes mellitus, type 1, Caregiver burden, Empowerment, Parents, Children, HbA1c

## Abstract

**Background:**

Parents play a critical role in caring for their children with diabetes and bear a significant burden of care. Health education has increasingly focused on empowering parents through new strategic methods. The present study aims to investigate the impact of a family-centered empowerment model on the burden of care in parents and blood glucose levels of children with type I diabetes.

**Methods:**

An interventional study was conducted in Kerman, Iran, where 100 children with type I diabetes and their parents were randomly selected as participants. The study aimed to implement a family-centered empowerment model in the intervention group, which consisted of four stages (educational phase, increasing self-efficacy, improving self-confidence, and evaluation) over a period of one month. The control group received routine training. The Zarit Caregiver Burden questionnaire and HbA1c log sheet were utilized to evaluate the effectiveness of the intervention. Questionnaires were administered before, after, and two months post-intervention, and data were analyzed using SPSS 15. Non-parametric tests were employed, and statistical significance was set at p < 0.05.

**Results:**

Before the study, no significant differences in demographic variables, level of burden of care, or HbA1C levels were observed between the two groups (p < 0.05). After the intervention, the burden of care score in the intervention group was significantly lower than that in the control group, both immediately post-intervention and two months later (P < 0.0001). Additionally, the median HbA1C levels in the intervention group were significantly lower than those in the control group after two months (6.5 in the intervention group and 9 in the control group) (P < 0.0001).

**Conclusion:**

The findings of this study suggest that the implementation of a family-centered empowerment model is an effective strategy for reducing the burden of care on parents of children with type 1 diabetes and for controlling the HbA1C levels of these children. Based on these results, it is recommended that healthcare professionals consider incorporating this approach into their educational interventions.

## Introduction

Type 1 diabetes is the most prevalent chronic metabolic disorder in children, affecting approximately one in every 300,000-500,000 children under the age of 18. Its prevalence is increasing in both children and adolescents [1]. The researchers have estimated that the prevalence of diabetes is 6% in the Iranian population, and it is estimated that around 4 million people are suffered from diabetes [2].

The diagnosis of diabetes in children can be a highly stressful experience for parents. The management of diabetes in children requires a strict and complex regimen, which includes continuous monitoring and attention from caregivers, particularly mothers [3]. This meticulous care schedule, combined with the negative effects of diabetes on family relationships, can impose a heavy burden on parents. Parents of children with diabetes may struggle to balance their child’s care with other needs and experience increased levels of stress, discomfort, family conflict, and burden of care [4].

Caregiver burden refers to a state of mental, physical, and social stress that can result in a range of problems, including burnout, anxiety, and depression [5]. There are two types of caregiver burden: objective and subjective. Objective burden refers to the time and financial resources required to provide care. Subjective burden, on the other hand, encompasses the physical, mental, social, and emotional pressures experienced by caregivers while performing their caregiving duties [6]. Prolonged periods of caregiving can lead to limitations in social life, uncertainty about care needs, constant worries, a heavy sense of responsibility, and an increased prevalence of depression among caregivers [7]. The burden of caring for a child can cause parents to experience burnout [8].

Research indicates that patients who actively participate in their disease therapy tend to achieve better treatment outcomes than those who are passive recipients of care [9], Empowerment training can promote disease awareness, increase motivation for self-care, and serve as a viable alternative to blindly following physician advice [10]. According to the World Health Organization, education is the cornerstone of diabetes treatment. Patient empowerment enables individuals to acquire the necessary knowledge and skills to manage their illness and make informed decisions about their care. Empowerment-based interventions not only identify patients’ challenges but also help them discover and utilize their full potential to manage and overcome diabetes [11]. The empowerment process involves three key concepts: knowledge and information, behavioral skills, and responsibility [12]. Mikhael et al. conducted a systematic review examining the effects of educational programs on self-management for patients with diabetes. The results of twelve studies showed that educational programs led to improvements in self-management behavior, knowledge, self-efficacy, health beliefs, and quality of life [13]. Similarly, Rezai et al. (2017) investigated the impact of a family-centered empowerment model on treatment adherence for patients with diabetes. Their findings suggested that this model could be beneficial in managing patients with diabetes as it improved knowledge and attitudes towards diabetes management [14].

Parents of children with diabetes often find it challenging to accept that their children must take insulin for the rest of their lives. They fear for their children’s future and wish to prevent them from having to take insulin, but unfortunately, there is no other alternative. Parents must support their children in leading healthier lives and controlling their blood sugar levels to avoid the severe consequences of diabetes in the future [15]. It is understandable that parents may struggle with knowing how to support their children with diabetes. Empowerment-based interventions are essential for patients with chronic diseases, and many diabetes experts believe that diabetes self-care is the primary responsibility of individuals and their families. Patients should control their diabetes in the most effective way possible, taking into account their cultural and personal backgrounds. As the most fundamental unit of society, the family has a responsibility to provide the patient and those around them with adequate and appropriate healthcare. As the majority of care is provided at home, the family’s role in supporting patients with diabetes to adapt to the stressors produced by the condition cannot be overstated [16]. Caregivers of children with type I diabetes often spend a significant amount of time providing care, which can result in fatigue and caregiver burden [17]. Based on observations and experiences, both children and parents desire to participate in self-care, but they may not know how to do so effectively, leading to significant challenges while caring for their sick children. As a result, researchers in a recent study aimed to investigate the impact of empowering children and families in caring for a child with type I diabetes on the caregiver burden experienced by parents and the child’s blood glucose levels.

## Methods

### Design

The present educational intervention study was conducted over a period of nine months, from October 2021 to June 2022, in Kerman province, Iran.

### Study setting

The research took place in clinics and the endocrinology department of diabetes hospitals affiliated with Kerman University of Medical Sciences. Kerman province is the largest province in Iran, characterized by a vast geographical distribution, attracting patients from various parts of the province and neighboring provinces. However, due to non-compliance with health guidelines and frequent visits, many of these patients do not have controlled blood sugar levels.

### Eligibility criteria

All children with type I diabetes and their parents who met the inclusion criteria were selected by random sampling.

Inclusion criteria were patients aged 6–14 years old with confirmed type I diabetes (fasting sugar above 126 mg/dc) for at least 2 months [18] who lived with their parents in Kerman and who had not participated in any other official diabetes-related programs other than routine hospital training in the previous month.

For individuals to be deemed eligible for inclusion in the study, it was necessary that parents had access to a smartphone with WhatsApp software installed, proficiency in the Persian language, and the ability to operate a smartphone.

The study’s exclusion criteria encompassed children with diabetes who had an additional chronic or acute condition, such as asthma [1], and family members who were part of the healthcare team [9]. The rationale behind excluding patients with other diseases was based on the study’s focus on the empowerment of diabetic patients, as co-morbidities could potentially introduce confounding factors. Hence, such patients were excluded from the study.

Furthermore, if parents failed to complete the questionnaires, or if 10% of the questions were left unanswered, they would be excluded from the study.

### Interventions

In order to conduct the research, the researcher visited the research settings, provided a detailed explanation of the study’s purpose to the participants and obtained written informed consent from parents and patients aged seven years and older, based on their level of understanding. In instances where parents were hesitant to participate in the study, the objectives were further clarified to ensure cooperation. Additionally, as an incentive for children’s participation, age-appropriate gifts were provided.

The parents were randomly assigned to either the control or the intervention groups. Prior to the intervention, parents completed demographic characteristic and Zarit Burden questionnaires. The research team, consisting of a PhD nurse from the medical-surgical and a PhD nurse from pediatric departments, and a MSN nurse, met the parents and their children in the clinical setting. During two one-hour sessions, the research team explained the study’s purpose and provided guidance to parents in the intervention group on how to access and view specific files, which were tailored to their children’s needs. Additionally, the team provided instructions on when and how to show the films to their children, and how to contact the researchers should any questions or concerns arise.

In this study, WhatsApp was utilized as a means of delivering instructional cartoons and files to both children and parents. These files were sent over a period of one month. Educational files were specifically designated for parents and sent on Friday mornings, allowing ample time for review prior to the next session. In addition, previous session content was summarized, enabling parents to focus on their child’s specific needs with the opportunity for private consultation or the delivery of a specialized video for individualized guidance.

The Alhani empowerment model was employed by the researchers which consists of four key components: (1) perceived threat, (2) skill and self-efficacy achievement, (3) increasing self-confidence through educational participation, and (4) evaluation [19]. The overarching aim of the study was to empower parents by increasing their knowledge and skillset in the care of children with diabetes. This approach was deemed essential for effective management of the disease.

In addition to routine training, the research team created educational pamphlets and videos that were approved by a pediatric endocrinologist for enhancing the knowledge of the intervention group.

During the first week of the intervention, the researchers provided information on diabetes, including its causes and contributing factors, as well as early and late complications. Additionally, the team discussed symptoms of both hypoglycemia and hyperglycemia, drug interactions, measures to take in times of danger, and how to calculate the appropriate insulin dose during periods of high blood sugar. Furthermore, the researchers provided guidance on the first center to contact in the event of any diabetes-related problems.

During weeks two of the intervention, the researchers focused on educating the intervention group about the diabetic diet and its impact on blood sugar control. Specifically, the team provided information on the carbohydrate content of various foods and the importance of carb counting during each meal. In weeks three and four, the researchers taught parents how to use a glucometer to assess blood sugar levels, how to draw and store insulin, appropriate injection techniques, and how to provide self-care. Additionally, the team discussed the importance of frequent and periodic visits, the benefits of walking and regular exercise, and how physical activity can help lower blood sugar levels.

To facilitate learning for children, instructional cartoons were also provided during the intervention. In order to increase self-efficacy among parents, a video was created to demonstrate correct insulin injection techniques. Additionally, parents were encouraged to improve their self-confidence by acting as health liaisons for their children and discussing diabetes-related issues with them. Finally, the effectiveness of the intervention was evaluated through sessions focused on empowerment, knowledge acquisition, and self-efficacy.

The assessment of knowledge was conducted at the beginning of each session, whereby parents were asked to respond to two oral questions related to the previous session’s content, while children were asked to describe the previous film or answer one question based on their age. Following the assessment, new content was presented. To evaluate self-efficacy, the researchers requested that both parents and children demonstrate or perform a skill via video call.

In the control group, parents and children received routine training provided by hospital personnel. Caregiver burden was measured for both intervention and control groups prior to, immediately following, and two months after the intervention. Additionally, HbA1C levels were measured before and two months after the intervention, with the analyzer remaining blinded to the participants’ group assignments.

### Sample size

In the present study, the sample size was estimated using a combination of the sample size formula and previous relevant studies [11]. The required sample size for this research was determined to be 50 for each of the control and intervention groups while ensuring that the groups were matched for age, sex, and laboratory testing location.

To recruit participants, the researchers visited several clinics and the endocrinology department of diabetes hospitals affiliated with Kerman University of Medical Sciences in Iran. Using a lottery method, the researchers randomly selected 100 children diagnosed with type 1 diabetes from checklists in the clinics, and subsequently assigned 50 children to each group. On days when parents brought their children to the ward, the research team interacted with parents and children, and enrolled them into either the control or intervention group if they expressed interest and were satisfied with the study requirements.

$$n = \frac{{{{\left( {{z_{1 - \frac{\alpha }{2}}} + {z_{1 - B}}} \right)}^2}\left( {d_1^2 + d_2^2} \right)}}{{{{\left( {{\mu _1} - {\mu _2}} \right)}^2}}}$$

$$n = \frac{{{{\left( {1.96 + 0.85} \right)}^2}\left( {15.3 * 15.3 + 18.35 * 18.35} \right)}}{{{{\left( {47.2 - 40.4} \right)}^2}}}$$

97.4738=

#### Measurements

In this research, data collection tools comprised of a demographic information questionnaire, the Zarit burden of caring caregivers questionnaire, and the HbA1c log sheet.

A) **Demographic information questionnaire**: The demographic information questionnaire included items related to the child’s age, father’s age, mother’s age, child’s weight, duration of illness, admission numbers, sex, birth rank, education level of the child, education levels of parents, occupations of parents, and marital status of parents.
B) **The Zarit burden of caring caregivers questionnaire**: This 22-item questionnaire was designed by Zarit et al. in 1986 to determine the extent of the burden. The validity and reliability of this questionnaire were confirmed in previous studies. The reliability of this questionnaire was confirmed using the retest method (0.94) and its validity, in addition to content validity, was confirmed using the Hamilton anxiety rating scale (r = 0.67) and Beck’s depression inventory (r = 0.89) [16]. Each question was rated on a 5-point Likert scale from zero (never) to four (always) with the sum of scores ranging between 0 and 88. A score of 0–20 was considered no burden, 21–40 was considered a moderate burden, 41–60 was considered moderate to severe burden, and 61–88 was considered a severe burden. The reliability and validity of the Persian version of this questionnaire were confirmed by Rajabi-Mashhadi et al. (2015), who conducted a study on the wives of veterans with chronic spinal cord injuries. The internal consistency of the questionnaire was strong (Cronbach’s alpha of 0.77). The correlation matrix between different domains of the questionnaire in the re-intervention was 0.78. The results of this study showed that the Persian version of the Zarit burden interview was valid and reliable for measuring the caregiver burden of people with chronic spinal cord injury [20].

##### The HbA1c log sheet

the researchers prepared the HbA1c log sheet to record the HbA1c level**s** before and two months later to measure.

#### Data analysis

SPSS15 was used to analyze the data. Median (interquartile range), standard deviation ± mean, frequency and percentage were used to describe the results. Kolmogorov-Smirnov test was used to measure the normality of data distribution. The data did not follow a normal distribution. Chi-square, Fisher’s exact, Mann-Whitney U, and Wilcoxon tests were used to analyze the data. The significance level was considered p < 0.05.

### Ethical consideration

This research was conducted after the acquisition of the code of ethics No.IR.KMU.REC.1400.225 from the ethics committee of Kerman University of Medical Sciences as well as the acquisition of the necessary permission from the university and diabetes center officials and the consent of the patients. All methods were performed in accordance with the relevant guidelines. The research units were assured that their participation in the study was voluntary and would not affect the provision of services and care to them. In addition, the necessary information and explanations about the research and the questions of the questionnaires were given to the participants, and they were assured that their information would be kept confidential. In addition, the researchers took informed consent from the parents of patients below 16 years.

## Results

100 children with their respective parents participated in this study. The mean age of the children was 9 years, while the mean age of the parents was 33 years. Among the children, a majority (56%) were girls.

The study findings indicated that both the control and intervention groups were comparable in terms of various demographic variables such as sex, age, weight, birth rank, education level, duration of illness, and admission numbers of the child, age of parents, education level of parents, occupation of parents, and marital status of parents. There was no statistically significant difference in these demographic variables between the two groups (P < 0.05), indicating homogeneity between the groups.

Table 1 showed a statistically significant difference in the within-group comparison of the burden scores in the control group before, after the intervention, and two months later, so routine training had a significant effect on reducing the burden scores of the parents (P < 0.04). In addition, there was a statistically significant difference in the within-group comparison of the burden scores in the intervention group before, after the intervention, and two months later, so the empowerment training played an effective role in reducing the burden scores. Therefore, 50% of the participants reduced severe burden to moderate burden after the intervention and to no burden two months after the intervention (P < 0.0001). Between-group comparison showed a statistically significant difference in burden scores between the control and intervention groups, so the median burden score in the intervention group was significantly lower than that in the control group (P < 0.0001).

**Table 1:** Comparison of the burden scores of parents of children with type 1 diabetes in control and intervention groups before, after the intervention, and two months later

Variable	Control group (N = 50)			Intervention group (N = 50)			P-value
	First quartile	Median	Third quartile	First quartile	Median	Third quartile	
Parents’ pretest burden scores	62	64.5	67	61.75	64	66	0.161 ^‡^
Parents’ posttest burden scores	52	54	56	19	23	26	< 0.0001 ^‡^
Parents’ burden scores two months later	50	52	54.2	17.7	20	21	< 0.0001 ^‡^
P-value	< 0. 04^€^			< 0.0001^€^			

* Independent t-test, € Freidman test, ‡ Mann Whitney U test

Table 2 showed no statistically significant difference in the severity of the burden of parents of children with diabetes between the two groups before the intervention (P = 0.338).

**Table 2:** Comparison of the severity of burden of parents of children with type 1 diabetes in control and intervention groups before, after the intervention, and two months later

Burden measurement time	Group	No burden	Moderate burden	Moderate to severe burden	Severe burden	P-value
Pretest	Control group (N = 50)	(0) 0	(0) 0	(8) 4	(92) 46	0.338*
	Intervention group (N = 50)	(0) 0	(0) 0	(14) 7	(86) 43	
Posttest	Control group (N = 50)	(0) 0	(0) 0	(100) 50	(0) 0	< 0.0001^£^
	Intervention group (N = 50)	(34) 17	(62) 31	(4) 2	(0) 0	
Two months later	Control group (N = 50)	(0) 0	(0) 0	(100) (50)	(0) 0	< 0.0001 ^£^
	Intervention group (N = 50)	(62) 31	(36) 18	(2) 1	(0) 0	

* Chi-squared test, £ Fisher’s exact test

In the intervention group, 34% and 62% of the parents reported no burden and moderate burden, respectively, but none of them reported a severe burden after the intervention. In addition, 50 people of the parents in the control group reported a moderate burden after the intervention, which showed routine care, could reduce the severe burden to moderate.

62% and 36% of the parents reported no burden and moderate burden, respectively, but none of them reported severe burden two months after the intervention. A statistically significant difference in the burden of care was observed between the control and intervention groups after the intervention, and two months later, so the burden of the parents in the intervention group was significantly lower than that of the parents in the control group (P < 0.0001).

Table 3 showed no statistically significant difference in the within-group comparison of HbA1C levels in the control group before and two months later (P > 0.05). The results showed a statistically significant difference in the within-group comparison of HbA1C levels of the intervention group before and two months later, so the empowerment training had an effective role in reducing the HbA1C levels of the children (P < 0.0001). Between-group comparison showed no statistically significant difference in HbA1C levels between the control and intervention groups before the intervention (P = 0.942). However, a statistically significant difference in HbA1C levels was available, between control and intervention groups, two months after the intervention, so the median HbA1C in the intervention group was significantly lower than that in the control group (P < 0.0001).

**Table 3:** Comparison of HbA1C levels between children with type I diabetes in control and intervention groups referred to diabetes control centers in Kerman in 2021 before and two months after intervention

Variable	Control group (N = 50)			Intervention group (N = 50)			P-value
	First quartile	Median	Third quartile	First quartile	Median	Third quartile	
Pretest HbA1C level	8.4	9.1	9.4	8.67	9.1	9.4	0.942 ^‡^
HbA1C level two months later	8.3	9	8.9	6.2	6.5	7.1	< 0.0001 ^‡^
P-value	< 0. 1^€^			< 0.0001^€^			

**‡ Mann Whitney U test**, € Wilcoxon

## Discussion

This study aimed to investigate the effect of family-centered empowerment model on burden of care of parents and blood glucose levels of children with type I diabetes referred to diabetes control centers in Kerman in 202.

The results showed that the level of burden was high, indicating that parents who have diabetic children have a high level of burden. Luo et al.(2021) demonstrated that parents of children with diabetes bear a lot of the burden of care, and the burden has a negative impact on their quality of life [21]. Saßmann et al. stated that Parents of children with diabetes had higher emotional and physical burdens. Parents of younger children described higher burdens compared to the parents of older children [22].

The results showed that the level of burden was high, indicating that parents who have diabetic children have a high level of burden. Luo et al.(2021) demonstrated that parents of children with diabetes bear a lot of the burden of care, and the burden has a negative impact on their quality of life [21]. Saßmann et al. stated that Parents of children with diabetes had higher emotional and physical burdens. Parents of younger children described higher burdens compared to the parents of older children [22].

The study results showed that there was a statistically significant difference in the burden scores of parents in the intervention and control group before, after the intervention, and two months later, so the empowerment training had an effective role in reducing the burden scores of parents. Therefore, 50% of the participants reduced their severe burden to moderate burden after the intervention and no burden two months later.

Farahmandnia et al. (2017) showed that family psychoeducation could reduce the burden of care in families of children with type 1 diabetes. It also reduced treatment costs and length of stay and increased the quality of care at home. This study was consistent with the current study because it focused on the effect of education on reducing the burden of family care [23]. Shiouh-Chu et al. (2012) studied caregivers of patients with colon cancer in Taiwan and showed that education and social support had a very positive effect on the burden of care, health, and financial affairs of parents [24].

Studies suggest that caregivers’ lack of adequate understanding of the disease process is the main cause of concern, and they bear a significant burden of care. Iranian parents are accountable for the care of their children. Parents of sick children frequently have to alter the conditions and habits of their family life, as well as some of their roles and responsibilities. They are mentally affected by their children’s circumstances, and in addition to their economic and occupational responsibilities, they must also care for other children and preserve family bonds. Several studies have found an inverse relationship between caregiver’s health and the burden of care. In addition, caregivers experience severe emotional and physiological stress when caring for their patients, which leads to physical and mental health problems in them and makes them feel unhappy, depressed, anxious, and hopeless [24].

Katz et al. (2014) showed that family psychoeducation caused better control of blood sugar, and more involvement and attention of family members to the patient with diabetes [25]. The strength of the Ketz study (compared with the current study) was the larger sample size and the follow-up of the subjects for two years, which showed the effect of prolonged intervention. Although the sort of education in this study differed from that in the current study, the results of the two studies were consistent. In addition, Ekinici et al. (2012) showed that family psychoeducation could reduce the burden of care of the families [26]. The results of this study are also in line with the current study in terms of the effect of education on reducing burden of care.

Yildiz et al. (2017) studied the level of self-efficacy and burden of caregivers of cancer patients in Turkey and discovered that caregivers’ perceived burden increased as patients’ distress and uncontrollable symptoms increased. Caregiver’s burden and self-efficacy levels were moderate in this study [27].

In general, studies show that parents may face physical, social, and economic problems when caring for patients with various diseases. Therefore, healthcare professionals must be aware of the role of parents when caring for a sick child because caregivers can significantly influence childcare. Caregiving burden has a negative effect on parental life and reduces the quality of life of the caregiver, as well as the quality of care and the quality of life of the patient [28] .

The results showed a statistically significant difference in the level of HbA1C of the children in the intervention group before and two months after the intervention, so the empowerment training had an effective role in reducing the HbA1C level of the children. The level of HbA1C reflects the average blood sugar in the last 2–3 months. Balancing HbA1C is a therapeutic goal in reducing the risk of hypoglycemia in patients with diabetes. Children with diabetes and a higher HbA1C level need a longer duration of adequate blood sugar management. Several factors affect HbA1C, which are difficult to identify. People with high HbA1C levels have more problems in the future.

Cheraghi et al. (2015) demonstrated that empowering adolescents with diabetes and their caregivers in-home care can improve the management of blood glucose levels and reduce HbA1C levels [29]. Frost (2014) found that the cycle of behavior change and sufficient support improved adherence to diet and exercise, and thus HbA1C [30]. According to Aghili et al. (2013), people with high levels of education adapted better to personal and social concepts related to diabetes. They could manage diabetes [31].

Mamaghani et al. (2021) revealed that empowerment-based training and focus on clients’ strengths in solving their problems could increase self-efficacy and reduce HbA1c levels [32].

Various studies suggest that training programs increase the knowledge and awareness of caregivers, and change their attitudes in order to help patients control their blood sugar and diabetes levels. Furthermore, people around patients and caregivers will have a better quality of life and burden of care. Some studies supported our study and indicated that empowerment-based training with group problem-solving strategies was effective in controlling diabetes and the hemodynamic status of patients. In general, family-centered interventions can have a positive role in the effectiveness of quality of life, disease control, and satisfaction with the provision of care services, as well as in cost-effectiveness, if families’ roles and responsibilities are clear and transparent. Therefore, the family-centered empowerment model may improve patients’ self-efficacy, changes patients and caregivers’ lifestyles positively, adjusts the burden of caregivers, and improves diabetes management.

This study also had limitations. One of the limitations of this study was that the samples were only selected from one province and the researchers do not know whether they can generalize to the whole country or not. Another limitation was that some of the data was missed, and the accuracy of data could not be managed because the questionnaires were completed by self-report.

## Conclusion

The results of the current study showed that the family-centered empowerment model was effective in reducing the burden of care of parents of children with diabetes due to the nature of the intervention and inspiration of patients to be more independent and meet their own care needs. Nurses at hospitals and home care settings can use the results of this study because this educational model is a simple model that nurses can easily use in order to reduce the burden of parents and reduce the blood sugar of patients.

## Data Availability

The data are available upon request to the corresponding author after signing appropriate documents in line with ethical applications and decision of the Ethics Committee.

## Abbreviations

HbA1c: Glycosylated Hemoglobin, Type A1C
